# Integrated management of childhood illness in Rwanda: Impact of mentorship on the quality of care in Nyanza and Huye districts

**DOI:** 10.1002/puh2.170

**Published:** 2024-03-25

**Authors:** Alain Mirindi, Assumpta Mwali Kayinamura, Amedee Fidele Ndibaza, Liliane Uwamahoro, Dieudonne Ndatimana, Ferdinand Bikorimana, Christian Mazimpaka, Richard Kalisa

**Affiliations:** ^1^ IntraHealth International Kigali Rwanda; ^2^ Maternal Child and Community Health Division Rwanda Biomedical Centre Kigali Rwanda; ^3^ School of Public Health University of Rwanda Kigali Rwanda

**Keywords:** facility readiness, health posts, Integrated Management of Childhood Illness, mentorship, quality of care

## Abstract

**Background:**

In Rwanda, health posts (HPs) are intermediary primary care facilities that provide comprehensive primary care services to communities and are located at a reasonable walking distance from people's homes. We assessed the readiness of HPs for Integrated Management of Childhood Illness (IMCI) services and examined changes in the quality of care for IMCI services between districts that implemented IMCI mentorship program (Nyanza district) and Huye district which it did not.

**Methods:**

We conducted a prospective cohort study to assess whether there was change in the quality of IMCI care provided at 17 Nyanza HPs 1‐year after IMCI mentorship implementation. The readiness of HPs for IMCI was assessed across nine factors, resulting in *essential* (all factors) and *desirable* (less than seven factors) composite scores. Unpaired *t*‐tests were used to measure changes in IMCI quality.

**Results:**

The HPs with IMCI mentorship had an increase in mean desirable (0.7–0.89) and essential (0.61–0.78) composite scores compared to non‐mentored HPs in Huye. The nurses who received mentorship program had improved scores in factors like IMCI training, service package availability, register availability, supportive supervision, and basic equipment availability. Quality improvements in IMCI assessments were observed in vital sign registration, danger sign detection, cough identification, malnutrition screening, and tuberculosis sign identification in the mentored HPs.

**Conclusion:**

Mentorship of nurses in HPs holds promise for enhancing facility readiness and IMCI service quality. Before expanding clinical mentorship, identified gaps such as staffing, supply chains, and health financing need to be addressed.

## Graphical abstract

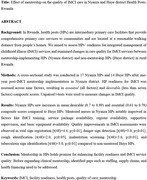

## INTRODUCTION

Almost three decades ago, the World Health Organization developed a strategy to address the major causes of global mortality in children under age five, known as Integrated Management of Childhood Illness (IMCI) [1, 2]. IMCI is an algorithmic symptomatic approach to pediatric visits designed to improve case management, health systems support, and family and community practices in lowest level health facilities [3]. Elsewhere authors have demonstrated that IMCI improved healthcare worker performance and quality IMCI which consequently lead to a reduction of government health expenditure and under‐five mortality [4, 5, 6]. Many countries still experience significant barriers such as inadequate infrastructure/equipment, shortage of staff, and political and financial constraints. Even in areas where IMCI has been implemented, there is still suboptimal clinical care [6, 7, 8] due to persistent challenges required to achieve and maintain associated benefits to quality IMCI‐related services [5, 8].

With this evidence in mind in 2006, the Rwanda Ministry of Health (MOH) began adopting, training, and implementing the IMCI protocol and by 2008 had scaled it in preservice training for nursing schools. This was proceeded with the initiation of support supervision by district hospital‐based supervisors who provided in‐service support to nurses at the health centers. However, due to other competing demands, these supervisors were unable to effectively provide on‐site clinical mentorship and support supervision but left with data collection and reporting [9, 10]. For last decade, MOH has been implementing low‐dose high frequency (LDHF) mentorship to address the previous challenges [11]. With this LDHF approach, a mentor visits health facilities for 2 days per month to work with the mentee on identified gaps and address them together. The mentor is equipped with observation checklists and skills assessment guides to evaluate mentee competencies which are aligned toward reduction of maternal, newborn, and child morbidities and mortalities [9, 11].

Rwanda's fourth health sector strategic plan outlines ambitious measures to ensure universal accessibility (in geographical and financial terms) of equitable and affordable quality health services (preventative, curative, rehabilitative, and promotional services) for all Rwandans [12]. Key to this goal is moving to a health post (HP) management model, a framework to streamline healthcare delivery, establishment, and functionality of HPs at the grassroots level [13]. They are intermediary primary care facilities located at the cell level which is reasonable walking distance from people's homes. [14] It can be anticipated that programs to scale up the implementation of quality pediatric services at HPs will preferentially benefit those in the lowest wealth quintiles and those who live in the rural areas [13, 15].

We evaluated the service readiness of HPs in the provision of IMCI services and compare changes in the quality of IMCI services from HPs implementing IMCI mentorship (from Nyanza district) to those HPs that did not (from Huye district) in rural Rwanda.

## METHODS

### Study design, setting, and population

We conducted a prospective cohort study to assess whether there was change in the quality of IMCI care provided at 17 Nyanza HPs 1‐year after IMCI mentorship implementation. We compared the difference in the quality of IMCI care provided at Nyanza HP to 14 Huye HPs that had nonexistent IMCI mentorship. Nyanza and Huye districts are both located in the southern province of Rwanda. Majority of residents from two districts were female in reproductive age and took mean walking distance of 57.5 min to reach nearby health center and 30 min to HP [14].

### Study variables and data collection

The HP's readiness assessment was analyzed through the lens of the nine factors adapted from the national guidelines for the establishment and functionality of HPs in Rwanda [12] (Table 1). The maximum score for the *essential* and *desirable* composite scores was one with each of the nine factors: staffing, supportive supervision, clinical care package, staff trained on IMCI, availability of IMCI treatment guidelines/tools, medical equipment, infection prevention and control (IPC) supplies and materials, laboratory services, and monitoring and evaluation information use. In terms of readiness, HPs identified with all the above factors were labeled *essential* and less than seven factors as *desirable*. Availability of IMCI treatment guidelines/tools, IPC materials/supplies, and laboratory services were directly observed. These factors received a score of 1 (one) if they were available, relevant, and functional. Laboratory services needed to be available, with unexpired reagents and/or functional to receive a score of one. Medical equipment needed to be available and functional to receive a score of one. The number of staff, staff trained on IMCI, HP service package, and strategic information and reporting were reported data elements obtained from the HP manager.

**TABLE 1:** Median desirable and essential composite scores for Nyanza and Huye district health posts readiness of Integrated Management of Childhood Illness (IMCI) services, Rwanda, 2022.

Composite Score	Nyanza	Huye
***Desirable***	***0.89***	***0.67***
Staffing	0.97	0.93
Health service package	0.97	0.89
Availability of basic medical equipment	0.75	0.63
Laboratory tests	0.91	0.86
Infection prevention and control	0.76	0.76
Availability of registers	1.00	0.21
Staff IMCI training	1.00	0.43
Supervised by the health center	1.00	0.71
Strategic information and reporting	0.65	0.57
***Essential***	***0.78***	***0.56***
Staffing	0.69	0.67
Health service package	0.82	0.79
Availability of basic medical equipment	0.78	0.65
Laboratory tests	0.88	0.71
Infection prevention and control	0.82	0.59
Availability of registers	0.76	0.33
Staff IMCI training	1.00	0.43
Supervised by the health center	0.79	0.36
Strategic information and reporting	0.50	0.50

**FIGURE 1:**
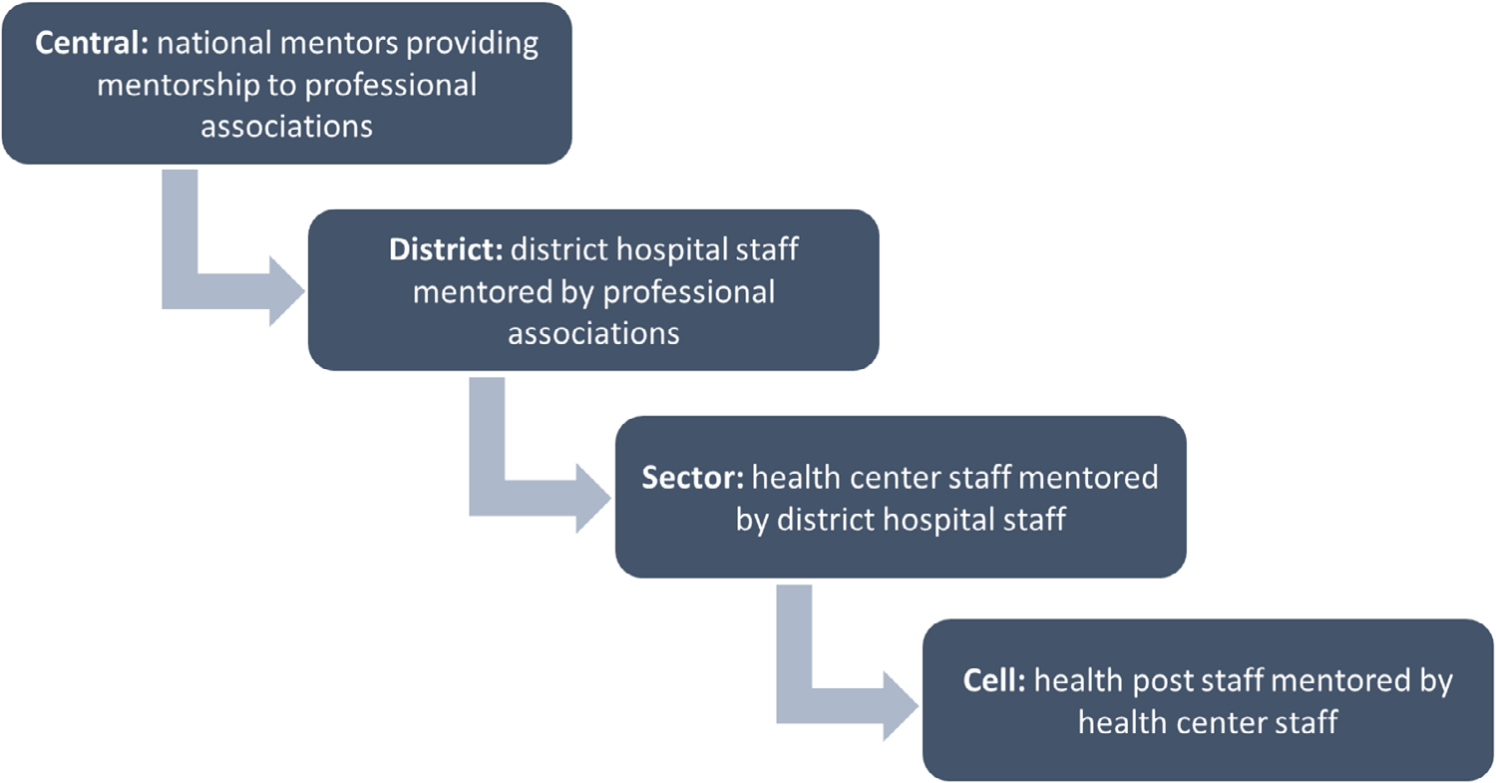
Rwanda step‐down clinical mentorship [11].

**FIGURE 2:**
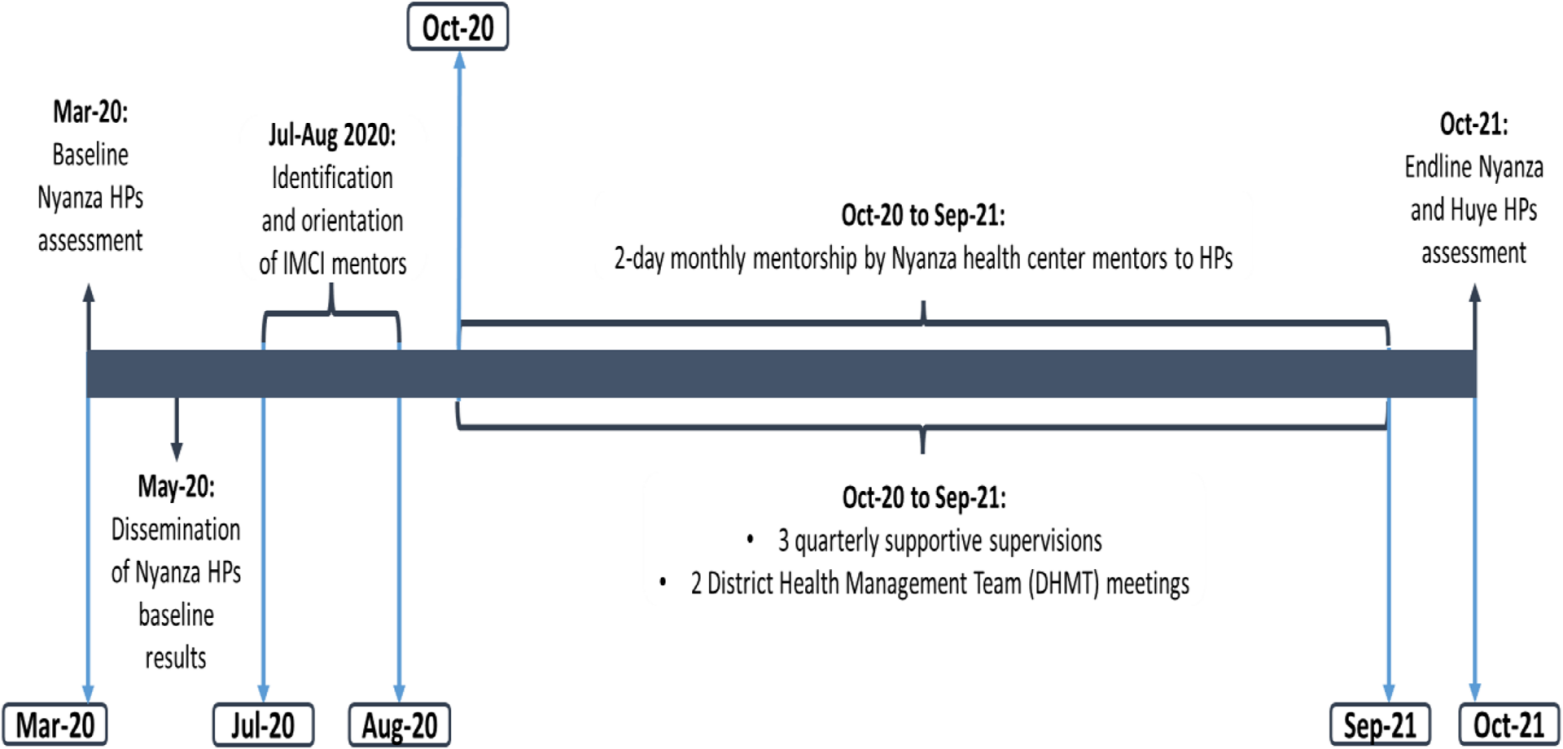
Implementation of Integrated Management of Childhood Illness (IMCI) mentorship and supportive supervision activities, Nyanza district health posts, Rwanda, 2020–2021.

Between October 2020 and October 2021 every month, a visiting nurse (*mentor*) from a health center within the HP catchment area visited an HP to provide a 2‐day IMCI mentorship to a nurse (*mentee*) providing IMCI services in Nyanza HPs. During that particular visit, the mentor would build the capacity of the IMCI mentee on identified mentees’ competency gaps as detailed in Table 2 so as to improve the quality of IMCI services provided to sick children. In addition, the HPs benefited from quarterly supportive supervision conducted by staff from the USAID Ingobyi Activity, health center, and Nyanza district hospital supervisors. Findings from supportive supervision visits were shared with the officer in charge of the HPs, the supervising health center, and Nyanza hospital administration and further disseminated in the district health management team (DHMT) and Nyanza district hospital coordination meetings (Figure 1, 2).

**TABLE 2:** Coverage and quality of Integrated Management of Childhood Illness (IMCI) provided at mentored (Nyanza) and non‐mentored (Huye) health posts, Rwanda, 2022.

	Mentored health posts	Non‐mentored health posts	*p* Value
Number of child visits observed	26	24	
***Assessment***			
Weighed	23 (88.46)	13 (54.17)	< 0.05
Temperature taken	23 (88.46)	15 (62.5)	< 0.05
Checked for three danger signs	22 (84.62)	12 (50)	< 0.01
Checked if the child is vomiting	22 (84.62)	11 (45.83)	< 0.05
Checked for the presence of convulsions	21 (80.77)	11 (45.83)	< 0.05
Checked for the presence of cough	18 (69.23)	12 (50)	0.166
Checked for the presence of diarrhea	2 (7.69)	4 (16.67)	0.329
Checked for the presence of fever	17 (65.38)	11 (45.83)	0.164
Vaccination status checked	17 (65.38)	12 (50)	0.271
Checked for anemia	23 (88.46)	10 (41.67)	< 0.001
Weight checked against a growth chart	16 (61.54)	6 (25.0)	< 0.01
Feeding practices assessed	3 (11.54)	10 (41.67)	0.053
Checked for other problems	21 (80.77)	19 (79.17)	0.887
***Treatment***			
The child correctly treated for diarrhea	1/1 (100.0)	1/2 (50)	
Child correctly treated for malaria	1/1 (100.0)	0/0 (NA)	
The child was correctly treated for pneumonia	2/2 (100.0)	1/1 (100.0)	_
***Counseling and communication***			
Caregiver advised on how to feed the child during illness	19 (73.08)	14 (58.33)	0.272

### Data analysis

We used essential and desirable composite scores and component scores for Nyanza and Huye HPs to compare readiness to provide IMCI services, calculated based on criteria for strengthening HP functionality using the national guidelines for the establishment and functionality of HPs in Rwanda [12]. The maximum score for essential was having all factors and desirable with less than seven factors. Nonparametric measures were used to compare median essential and desirable composite scores using Microsoft Excel (Version 15.0).

To evaluate the quality of IMCI provided by nurses from mentored HPs (Nyanza district) and non‐mentored HPs (Huye district), we compared child assessment, classification, and diagnosis findings provided in routine IMCI services. To assess the performance of the two districts’ IMCI nurses, the proportion of observed children correctly assessed for each main symptom was calculated for each IMCI nurse. Using the health outcomes of children as the unit of analysis, we then calculated the proportion of children with each main symptom who were assessed correctly, assessed incorrectly, or not assessed at all.

### Ethical considerations

All study participants provided signed informed consent in the local language (Kinyarwanda) prior to data collection. We received ethical approval from the Rwanda National Ethics Committee (Kigali, Rwanda, No: 48/RNEC/2022) as well as from IntraHealth International's Institutional Review Board (Chapel Hill, North Carolina, 27517, United States, No: 21006).

## RESULTS

Comparing the overall composite score, where mentorship was conducted, HPs’ readiness (Nyanza district) was significantly different compared to HPs without mentorship (Huye district) with median desirable composition scores of 0.89 versus 0.67 as well as in essential composite scores of 0.78 versus 0.56, respectively (Table 1).

The majority of the improvements in both desirable and essential composite scores were accounted for by increases in the health service package, availability of laboratory services and IMCI registers, regular supportive supervision by health centers, and staff being trained on IMCI (Table 1).

The majority of the HPs already had at least four of the five essential equipment components including a weighing scale/height board, mid‐upper arm circumference tapes, thermometer, otoscope/stethoscope, and pharmacy. In terms of desirable (0.75 vs. 0.63) and essential (0.78 vs. 0.65) component scores, significant improvement was achieved where mentorship took place as compared to non‐mentored HPs (Table 1).

The largest improvements were seen in both desirable (0.91 vs. 0.86) and essential (0.88 vs. 0.71) laboratory service components from mentored and non‐mentored HPs (Table 1).

The majority of HPs were understaffed with desirable and essential component scores declining in both mentored and non‐mentored HPs. However, in Nyanza HPs, at least those few nurses had benefited from existing IMCI mentorship as compared to those in Huye who did not (Table 1).

There was an improvement in the provision of regular IMCI services with the desirable component score increasing to 0.97 at mentored HPs in Nyanza versus 0.89 at non‐mentored HPs in Huye and the essential component increasing to 0.82 versus 0.79 (Table 1).

Improvement in either desirable or essential M&E/information use, reporting, and availability for IMCI registers occurred more in mentored HPs than non‐mentored HPs between baseline and end‐line (Table 1).

There was significant change in quality of IMCI assessments among children who had been managed by mentored nurses with improvements in vital signs registration [*t*(48) = 4.4, *p* < = 0.01)], danger signs [*t*(48) = 3.0, *p* < = 0.01)], fever [*t*(48) = 2.0, *p* < = 0.05)], and screening for malnutrition [*t*(48) = 3.6, *p* < = 0.01) as compared to HPs without mentorship (Table 2).

## DISCUSSION

Our study revealed that even the lowest level healthcare facilities such as HPs can provide high‐quality child health services when adequately equipped and supported through IMCI mentorship. Essential prerequisites for such effective implementation encompass the provision of sufficient drugs/supplies, necessary equipment, conducive support supervision, and administrative advocacy by the DHMT and district hospital managers for equitable service delivery. Such comprehensive preparedness across HPs could potentially result in heightened coverage and superior quality of IMCI services [16, 17].

Our findings identified an uptick in scores related to factors such as healthcare package, IMCI staff training, availability of medical equipment, and laboratory tests. In contrast, other parameters like nurse availability, support supervision visits, and the supply of IPC materials demonstrated comparatively slower progress during the IMCI implementation phase. This disparity could suggest a focus on certain aspects over others during the mentorship period. The COVID‐19 crisis which necessitated the redeployment of limited staff for pandemic‐related activities could have further affected routine activities including supportive supervision [18, 19, 20].

Contemplating improvement strategies for child health services at primary points of care like HPs, it becomes imperative to explore the “how” of an intervention [21]. This aspect is both central and context‐specific. Therefore, reflecting on the nuances of the mentorship implementation strategy, adjustments made, and learnings accrued, we organized quarterly meetings with DHMTs and district hospitals. In these meetings, implementing partners and district leadership fostered a conducive environment for data‐driven decision‐making, enhancing component scores for drugs, supplies, and equipment and integrating HPs’ service package into accreditation standards for easier reimbursement by the Rwanda Social Security Board. This interconnected commitment facilitated health systems managers in bridging these gaps equitably via targeted resource allocation [18].

Our study witnessed a significant improvement in IMCI assessment, classification, and treatment quality indicators in mentorship‐inclusive HPs. Key assessments included vital signs registration, danger signs, cough, malnutrition screening, and tuberculosis signs identification. Given the current shortage of nurses, our findings strongly advocate for scaling up IMCI mentorship leading to improved early detection of emergency cases and timely referrals. Such a step could potentially contribute to reducing infant and under‐five mortality rates. This necessitates robust investment from the private sector and nonprofit stakeholders to address domains like staffing, supply chains, and health financing for optimal HP performance.

This is one of the first few studies in Rwanda to assess HP facility readiness geared toward improved quality of IMCI services. The strength of the study lies in its adherence to national guidelines for measuring facility readiness [12]. However, the complexity of facility‐level decision‐making and other possible explanations for the decline in certain factors and closure of some HPs were not evaluated. These limitations warrant further exploration.

## CONCLUSION

Mentorship within HPs presents a considerable opportunity to improve facility readiness and the quality of IMCI services. This HP readiness and quality of IMCI services were markedly low in HPs devoid of mentorship programs. Therefore, before considering an extensive implementation of clinical mentorship at HPs, it is crucial to address the identified obstacles, especially in staffing, supply chains, and health financing. A thorough resolution of these issues will likely pave the way for more IMCI coverage and efficient and higher quality IMCI services in more than 1700 newly established HPs in Rwanda.

## AUTHOR CONTRIBUTIONS

*Conceptualization; writing—original draft; methodology; formal analysis; writing—review and editing; Supervision*: Alain Mirindi. *Conceptualization; writing—original draft; methodology; validation; project administration*: Assumpta Mwali Kayinamura. *Writing—original draft; writing—review and editing; formal analysis; data curation*: Amedee Fidele Ndibaza. *Writing—original draft; writing—review and editing; methodology*: Liliane Uwamahoro. *Writing—original draft; methodology; writing—review and editing*: Dieudonne Ndatimana. *Writing—original draft; writing—review and editing; conceptualization; project administration*: Ferdinand Bikorimana. *Conceptualization; writing—original draft; writing—review and editing; methodology; project administration; validation*: Christian Mazimpaka. *Conceptualization; writing—original draft; writing—review and editing; methodology; project administration; supervision; validation*: Richard Kalisa.

## CONFLICT OF INTEREST STATEMENET

Authors declare that they have no conflicts of interest.

## FUNDING INFORMATION

No funding was received for this research. The study was conducted as an activity of USAID Ingobyi Activity of IntraHealth International in Rwanda.

## ETHICS STATEMENT

All study participants provided signed informed consent in the local language (Kinyarwanda) prior to data collection. We received ethical approval from the Rwanda National Ethics Committee (Kigali, Rwanda, No: 48/RNEC/2022) as well as from IntraHealth International's Institutional Review Board (Chapel Hill, North Carolina, 27517, United States, No: 21006).

## Data Availability

The datasets for the current study are available from the corresponding author on request.
